# Assessing the context of health care utilization in Ecuador: A spatial and multilevel analysis

**DOI:** 10.1186/1472-6963-10-64

**Published:** 2010-03-12

**Authors:** Daniel F López-Cevallos, Chunhuei Chi

**Affiliations:** 1 Division of Health and Physical Education, Western Oregon University, 345 N Monmouth Ave, Monmouth, Oregon, 97361, USA; 2 International Health Program, Department of Public Health, Oregon State University, 254 Waldo Hall, Corvallis, Oregon, 97331, USA

## Abstract

**Background:**

There are few studies that have analyzed the context of health care utilization, particularly in Latin America. This study examines the context of utilization of health services in Ecuador; focusing on the relationship between provision of services and use of both preventive and curative services.

**Methods:**

This study is cross-sectional and analyzes data from the 2004 National Demographic and Maternal & Child Health dataset. Provider variables come from the Ecuadorian System of Social Indicators (SIISE). Global Moran's I statistic is used to assess spatial autocorrelation of the provider variables. Multilevel modeling is used for the simultaneous analysis of provision of services at the province-level with use of services at the individual level.

**Results:**

Spatial analysis indicates no significant differences in the density of health care providers among Ecuadorian provinces. After adjusting for various predisposing, enabling, need factors and interaction terms, density of public practice health personnel was positively associated with use of preventive care, particularly among rural households. On the other hand, density of private practice physicians was positively associated with use of curative care, particularly among urban households.

**Conclusions:**

There are significant public/private, urban/rural gaps in provision of services in Ecuador; which in turn affect people's use of services. It is necessary to strengthen the public health care delivery system (which includes addressing distribution of health workers) and national health information systems. These efforts could improve access to health care, and inform the civil society and policymakers on the advances of health care reform.

## Background

Over the past few years Ecuador has slowly emerged from a deep political, economic, and social crisis that has had a heavy impact on all sectors, with vulnerable groups being the hardest hit. The main political and social problems that have a direct impact on the health situation include high levels of poverty, limited access to health services, and low health insurance coverage [1]. This is due in part to the lack of a national health care system structured as indicated in the National Constitution (which was recently revisited by the 2008 Constitutional Assembly). The health care sector in Ecuador is constituted by a mix of public and private providers. The majority (85%) of health care facilities operate under public institutions: the Ministry of Public Health (MPH), the Ecuadorian Social Security Institute (IESS), the Military and Police Health Services (under the Ministries of Defense and Government, respectively), and the health services of certain provinces and municipalities [2]. The Ecuadorian Congress approved in 2000 a health care reform law in order to establish a National Health Care System (NHCS). Although there are important pieces missing (such as how to finance Universal Health Insurance; connection with local and regional services, etc.), it provided a basic agreement to work on for the future of a NHCS. In its report about Health Reform in Andean Countries, Pan American Health Organization (PAHO) mentions that Ecuador has not shown evidence that health care reform influenced any indicators selected to evaluate access or use of health care resources, including distribution of services [3]. Moreover, the political instability of the past decade has caused problems in governance and continuity in public management, which in turn has affected the health sector reform process [4].

From a public health and ecological perspective, it is important to analyze contextual factors affecting the use of health services at the community, institutional and policy levels [5,6]. During the last 40 years, Andersen's Health Care Utilization Behavior model has been adapted to consider more system-level measures, focusing on the availability, organization and financing of services [7,8]. Further, these literature indicated that besides predisposing, enabling and need factors, the environment and provider-related factors also affect healthcare utilization [9]. From a programmatic and policy perspective, connecting peoples' perceptions of health services and health care delivery system characteristics can contribute to our understanding of utilization behavior in a more comprehensive manner. In a systematic review of the literature, Phillips et al. (1998) found that the majority of studies that included environmental variables measured only urban/rural location, or region, which may be imprecise proxies for more specific measures such as supply of services. Hence, characteristics such as *physician supply *and *availability of physicians in the community *would be important contextual variables to be considered within the health services utilization model [9,10]. There are few studies that have analyzed the context of health care utilization in Latin America. Most studies have focused on the relationship between income inequality and health outcomes [11-13]. However, advances in health geography have improved our understanding of the role played by geographic distribution of health services on access to health services [14,15]. Notably, a study in Costa Rica linked census data with an inventory of health facilities allowing the researcher to analyze the impact of reform expansions on equity in provision of health care services[16].

The purpose of the present study is to analyze the context in which utilization of health services in Ecuador takes place, focusing on the provision of services. More specifically, this inquiry is focused on two research questions: 1) What is the spatial distribution of health care providers at the province level in Ecuador?; 2) What is the influence of provider measures (adjusting for predisposing, enabling, and need factors) in use of health care services in Ecuador?

## Methods

### Data

The main dataset analyzed in this study was the 2004 Demographic and Maternal & Child Health Survey (2004 ENDEMAIN) [17]. Using a multistage clustering design, ENDEMAIN 2004 provided a nationally representative sample of 28,908 households in Ecuador. The 2001 National Census was utilized as the sampling frame for selecting individual non-institutionalized households within census sectors. Two separate questionnaires were applied to different sub-samples: 1) an interview with a woman of reproductive age about sexual and reproductive health issues was completed in 10,813 households; and 2) an interview with an adult about health utilization and expenditures of all household members was completed in 10,985 households. For the later, ENDEMAIN 2004 gathered information on utilization of health services, and health care and consumption expenditures. In this survey, the response rate was 88.7% [17,18]. Various provider measures at the province level were extracted from the Ecuadorian System of Social Indicators (SIISE, v4.0, 2005). All provider-level measures were tabulated by SIISE from the Health Care Resources Survey (ERAS) 2001 data. The number of outpatient clinics per 10.000 inhabitants is the only measure that is not disaggregated into public/private due to data constraints. Public health care services include those provided by: Ministry of Health, IESS, ISSFA, ISSPOL, province and municipality governments; while private health care services include non-for-profit and for-profit providers. Health personnel measures comprised dentists, obstetricians, nurses, and health aides. The provider measures included density of: a) public practice physicians, b) private practice physicians, c) public practice health personnel, d) private practice health personnel, e) public inpatient clinics, f) private inpatient clinics, g) outpatient clinics, per 10 000 inhabitants [19]. Table 1 presents a summary of province level measures.

**Table 1:** Summary statistics of province level measures.

Variable	n	Mean	SD
***Provider measures*^a ^**			
Public practice physicians	22	8.401	2.352
Private practice physicians	22	5.450	4.243
Public practice health personnel	22	26.044	16.179
Private practice health personnel	22	7.239	6.171
Public inpatient clinics	22	0.277	0.242
Private inpatient clinics	22	0.305	0.176
Outpatient clinics	22	3.919	1.762

^a ^Per 10 000 inhabitants.

Following the proposed research questions, this study includes two components. First, an ecological analysis of provider measures at the province level was conducted using both correlation and spatial clustering. The spatial analysis verified the distribution of provider measures across space. Second, a multilevel analysis of the relationship between provider measures and use of health services was conducted. Using 2004 ENDEMAIN, Andersen's model of health care utilization served as a framework to classify predictors of health care utilization in three categories: predisposing (demographic), enabling (socioeconomic), and need factors. *Predisposing factors *included age, sex, ethnicity, and marital status. ENDEMAIN 2004 asked for marital status of individuals over 12 years of age. We assigned the category *single *to these individuals (to capture young children's health care utilization). *Enabling factors *consisted of area of residence, assets quintile, consumption quintile, educational level, and health insurance status. Since ENDEMAIN 2004 asked for educational level of individuals 5 years of age and older, we assigned the category *none *to these individuals (to capture young children's health care utilization). In ENDEMAIN 2004, two indexes were created as a ranking (quintiles) of household economic status: 1) an economic index based on household characteristics and durable goods availability (*Assets quintiles*); and 2) an economic index based on household consumption of goods and services (*Consumption quintiles*). Further explanation on the development of both variables can be found at: http://www.cepar.org.ec/endemain_04/nuevo05/informe/anexos/anexo1.htm*. Perceived need *was defined as the reported number of health problems by respondents. Participants were asked for the two most important health problems during the previous 30 days. Health care utilization was measured by use of preventive services and curative care visits. Due to the way the survey was designed, curative visits were specific to each of the two health problems reported. Table 2 summarizes the predictors included in the analysis for use of preventive and curative care.

**Table 2:** Unweighted summary statistics for use of preventive and curative care.

				*Curative Care Visits*	
				
	Level	Sample (n = 46497)	Use of preventive care (n = 2539)^a^	First health problem (n = 8152)^a^	Second health problem (n = 904)^a^
***Predisposing factors***					

Age in years: mean (SD)	Individual	26.98 (20.67)	22.97 (21.24)	28.47 (23.65)	38.85 (26.05)
Sex (%)	Individual				
Male		49.50	42.69	46.69	41.70
Female		50.50	57.31	53.31	58.30
Ethnicity (%)	Household				
Mestizo		84.93	87.83	89.40	89.60
Indigenous		9.20	5.44	5.81	4.20
Others		5.87	6.73	4.78	6.19
Marital status (%)	Individual				
Living w/partner		11.15	8.31	10.38	13.27
Married		26.74	25.68	29.24	35.29
Separated/divorced		3.75	3.03	4.39	6.19
Widow		2.94	2.48	4.33	10.73
Single		55.41	60.50	51.66	34.51

***Enabling factors***					

Area of residence (%)	Census segment				
Urban		50.50	62.86	57.69	61.62
Rural		49.50	37.14	42.31	38.38
Assets quintile (%)	Household				
1		23.74	11.22	18.02	16.92
2		21.32	16.19	21.22	23.01
3		19.29	19.85	21.17	21.90
4		18.14	21.54	20.61	20.02
5		17.51	31.19	18.98	18.14
Consumption quintile (%)	Household				
1		24.84	14.14	19.09	17.26
2		21.27	14.81	20.82	21.02
3		19.57	18.75	21.07	20.91
4		18.22	24.22	20.73	23.12
5		16.10	28.08	18.29	17.70
Educational level (%)	Individual				
None		21.56	30.25	30.61	28.21
Elementary		44.77	30.33	40.42	44.47
High School		25.53	27.18	21.74	19.91
College		8.04	12.13	7.19	7.30
Doesn't know/answer		0.11	0.12	0.05	0.11
Insurance (%)	Individual				
Insured		19.46	26.94	21.88	25.11
Uninsured		80.54	73.06	78.12	74.89

***Need***					

Health problems (%)	Individual				
No problems		52.64	52.74	0.00	0.00
One problem		41.24	41.04	87.93	0.00
Two problems		6.12	6.22	12.07	100.00

^**a **^Subsamples highlight those individuals who answered "Yes" to use of preventive care services, first health problem curative visit, and second health problem curative visit, respectively.

### Statistical Analysis

The first part of the analysis included both a non-spatial measure of correlation (r) and a spatial measure of correlation (Global Moran's I statistic). The correlation matrix (Table 3) showed that density of outpatient services, public inpatient services, and private inpatient services were highly correlated with public practice physicians (r = 0.64), public practice health personnel (r = 0.70), and private practice physicians (r = 0.78); respectively. Therefore, further analyses included only the following four predictors: public and private practice physicians; and public and private practice health personnel.

**Table 3:** Bivariate Pearson correlation coefficients for provider measures.

	Outpatient Services	Public Inpatient Services	Private Inpatient Services	Public Practice Physicians	Public Practice Health Personnel	Private Practice Physicians	Private Practice Health Personnel
Outpatient Services [per 10 000 inhabitants]^a ^	1.000						
Public Inpatient Services	0.744***	1.000					
Private Inpatient Services	-0.496**	-0.472**	1.000				
Public Practice Physicians	0.644***	0.580***	-0.299*	1.000			
Public Practice Health Personnel	0.482**	0.695***	-0.373**	0.583***	1.000		
Private Practice Physicians	-0.487**	-0.513***	0.778***	-0.003	-0.295*	1.000	
Private Practice Health Personnel	-0.148	-0.055	0.121	0.150	0.360*	0.320*	1.000

* p < 0.1, ** p < 0.05, *** p < 0.01.

^**a **^All provider measures have the same denominator.

**Figure 1:**
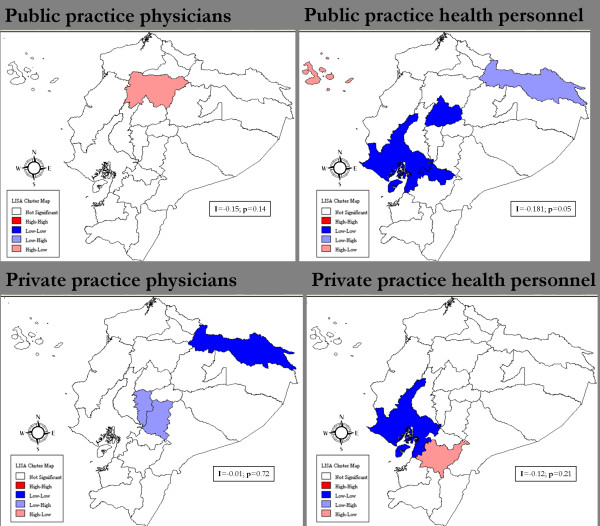
**LISA Cluster Maps of Provider Measures density by province**. The high-high (red) and low-low (blue) locations suggest clustering of similar values.

Global Moran's I statistic is a measure of spatial autocorrelation (SA), which is the correlation of a variable with itself through space (i.e. it compares the value of the variable at any single location with the value at all other locations). SA can assess any systematic pattern (clustering) in the spatial distribution of that variable (i.e. the two dimensional equivalent of redundancy) [20,21]. If neighboring areas are more similar, we would obtain a *positive *spatial autocorrelation which might violate the assumption that the observation values in each sample are independent of each other. A Moran's I value near +1.0 indicates clustering; 0 indicates randomness; and a value near -1.0 indicates dispersion. This analysis also produced univariate LISA (Local Indicator of Spatial Autocorrelation) maps to assess specific spatial patterns of provider variables (Figure 1).

The second part of the analysis used multilevel models to examine the relationship between provider level measures and individual health care utilization (adjusting for various relevant predisposing, enabling and need factors). The multilevel analysis was better suited to account for the four levels in ENDEMAIN sampling frame. The highest level consisted of 17 strata which included two regions (Amazon and Galapagos Islands) and 15 provinces (10 from Sierra region and 5 from Costa region). Consequently, the term province/region refers to this particular level of analysis. The four levels included: 1) 17 provinces; 2) 692 census segments; 3) 10 985 households; and 4) 46 497 individuals. In doing so, this study explicitly accounted for clustering in such a complex sample design [22-24]. Furthermore, multilevel modeling offered the possibility of merging provider measures at the province/region level to the analysis. Since ENDEMAIN 2004 reduced the *Oriente *provinces (Sucumbíos, Orellana, Napo, Pastaza, Morona Santiago, and Zamora Chinchipe) to a single stratum, an average was calculated for each *Oriente *provider measure to be included in multilevel models. Provider measures were linked with the ENDEMAIN dataset using the Stata merge command. After checking for accuracy of merging, the new dataset was imported into MLwiN for multilevel analyses. This is advantageous for our analysis given that the ENDEMAIN data does not include local level provider information. Given that 97% of private practice physicians are concentrated in urban areas (86% among public practice physicians) [19], the final conditional models included interaction terms between provider measures and the urban/rural dummy variable.

A common way of specifying multilevel models is to build them in a sequential manner [13], starting with a *non-conditional *(empty) model to partition variance across levels and assess its statistical significance [25]. These models are then estimated by the iterative generalized least squares maximum likelihood estimator. Because all individual-level outcome variables were dichotomous, we applied the marginal quasi likelihood approximation with a first order Taylor linearization procedure [13]. For each outcome variable, predictors were assigned to each significant level (as they were originally collected) to build various *conditional *models. For provider measures, preliminary bivariate multilevel analyses determined the variables significantly associated (at p < 0.1) with the outcomes of interest. Only these provider measures were utilized in further analyses. The final multivariate multilevel models were adjusted for predisposing, enabling, need factors, and interaction terms. For data preparation, descriptive statistics, and merging we used Stata MP v9.2 [26]. To calculate the Global Moran's I and LISA functions we used GeoDa 0.9.5-i5 [27]. Lastly, we used MLwiN 2.02 to fit all multilevel models [28].

## Results

Spatial autocorrelation (Moran's I) scores revealed no significant spatial clustering of provider measures by province. For public practice health personnel, although the initial Global Moran's I showed no clustering (I = -0.18, p < 0.05), it became somewhat clustered when the provinces of Galapagos and Sucumbíos (outliers) were excluded (I = 0.09, p < 0.05). Moreover, LISA analysis showed there was a cluster of low density of public practice health personnel in Guayas, Cañar, and Cotopaxi (provinces in blue, Figure 1). In summary, the preliminary ecological analysis showed that, for the most part, the provider measures were randomly distributed across space. In other words, in this study provider density seemed equally distributed across provinces in Ecuador.

Table 4 presents the final conditional multilevel models for use of preventive and curative care. Except for provider measures and age, all explanatory variables were entered in the models as indicator dummy variables. In bivariate analyses, three provider measures were significantly associated with use of preventive care (public practice physicians, public practice health personnel, and private practice health personnel). After the model adjusted for predisposing, enabling, and need factors, the density of public practice health personnel had a significant positive association with use of preventive care (OR = 1.015, 95%CI: 1.003-1.027). This result suggests that for a 1-unit increase in the density of public practice health personnel, the odds of using preventive care by Ecuadorians increased 1.5%. The final model included three interaction terms. The public practice health personnel*rural was the only statistically significant interaction term (OR = 1.018, 95%CI: 1.008- 1.028). In other words, density of public practice health personnel has a bigger impact among rural households, by increasing to 1.8% the odds of using preventive care (notice how *public practice health personnel *is no longer statistically significant when including the interaction term). This is an interesting finding, considering that initially there was no significant difference in use of preventive care between urban and rural households (OR = 0.92, 95%CI: 0.67 - 1.27). Both assets and consumption quintiles show a gradient where the poorest households are least likely to use preventive care (OR_assets _= 0.40, 95%CI = 0.32-0.50; OR_consumption _= 0.49, 95%CI = 0.40-0.60) in comparison with the wealthiest households (i.e. use of preventive services increases with wealth).

**Table 4:** Multilevel weighted regression estimates for use of preventive and curative care.

	Use of preventive care^a^	*Curative Care Visits*	
		
		First health problem^a^	Second health problem^a, b^
**Parameters**			

Constant	-2.161	-1.457	-4.470
***Provider measures***			
Public practice physicians	0.044 (0.035)		
Private practice physicians		0.034 (0.007)***	0.042 (0.013)***
Public practice health personnel	0.009 (0.006)	-0.015 (0.002)***	-0.018 (0.005)***
Private practice health personnel	-0.010 (0.009)		
***Predisposing factors***			
Age	-0.015 (0.002)***	0.003 (0.002)	0.017 (0.003)***
Female	0.314 (0.042)**	0.114 (0.030)***	0.273 (0.046)***
Indigenous	-0.238 (0.196)	-0.385 (0.080)***	
Other ethnicity	0.140 (0.074)*	-0.266 (0.077)***	
Living w/partner	0.024 (0.070)	-0.212 (0.074)***	-0.074 (0.113)
Separated/divorced	-0.182 (0.135)	-0.055 (0.057)	-0.004 (0.122)
Widow	0.223 (0.088)**	0.076 (0.062)	0.378 (0.146)**
Single	-0.217 (0.061)***	-0.281 (0.055)***	-0.347 (0.069)***
***Enabling factors***			
Rural	-0.079 (0.164)	-0.280 (0.086)***	-0.443 (0.127)***
Assets quintile 1	-0.918 (0.115)***	-0.059 (0.106)	
Assets quintile 2	-0.443 (0.100)***	0.114 (0.126)	
Assets quintile 3	-0.235 (0.068)***	0.147 (0.094)	
Assets quintile 4	-0.249 (0.053)***	0.143 (0.065)**	
Consumption quintile 1	-0.710 (0.100)***	-0.497 (0.056)***	
Consumption quintile 2	-0.585 (0.088)***	-0.295 (0.046)***	
Consumption quintile 3	-0.434 (0.072)***	-0.221 (0.052)***	
Consumption quintile 4	-0.182 (0.078)**	-0.100 (0.049)**	
No education	0.697 (0.081)***	1.211 (0.073)***	1.036 (0.201)***
Elementary school	-0.021 (0.074)	0.473 (0.054)***	0.460 (0.152)***
High school	0.033 (0.067)	0.184 (0.033)***	0.136 (0.157)
Doesn't know/answer	0.087 (0.572)	-0.218 (0.458)	0.741 (0.829)
Uninsured	-0.425 (0.059)***	-0.279 (0.033)***	-0.346 (0.072)***
***Need***			
One health problem	-0.038 (0.035)	N/A	N/A
Two health problems	0.113 (0.084)	N/A	N/A
***Interaction terms***			
Public practice physicians * Rural	-0.035 (0.025)		
Private practice physicians * Rural		-0.013 (0.007)*	-0.017 (0.013)
Public practice health personnel * Rural	0.018 (0.005)***	0.008 (0.003)***	0.006 (0.006)
Private practice health personnel * Rural	-0.010 (0.009)		
***Random parameters***			
Level 4: province/region	0.031 (0.016)*	0.016 (0.007)**	0.117 (0.086)
Level 3: census segment	0.081 (0.025)***	0.071 (0.012)***	0.161 (0.056)***
Level 2: household	2.351 (0.452)***	0.661 (0.062)***	1.941 (1.281)

* p < 0.1, ** p < 0.05, *** p < 0.01. N/A = Not applicable.

^**a **^A four-level binomial logit model (IGLS, MQL1) was run for each outcome. The final conditional model was fitted with health care need, predisposing and enabling factors as fixed effects, and random variance at the household, census segment and province/region levels. Interaction terms were included between the provider measures and urban/rural. Figures in parentheses represent the standard errors.

^**b **^Since no significant variation was found at the household level in the non-conditional model, no predictors at this level were included in subsequent models.

In bivariate analyses, two provider measures were significantly associated with both measures of curative care: private practice physicians, and public practice health personnel. The density of private practice physicians had a positive association with *first health problem curative visit *(OR = 1.027, 95%CI = 1.013-1.042), even after adjusting for predisposing and enabling factors. In contrast, the density of public practice health personnel was negatively associated with the outcome of interest (OR = 0.987, 95%CI = 0.983-0.991). Interestingly, the interaction terms showed almost the opposite. Among rural households, density of public practice health personnel was positively related to use of curative services for the first reported health problem (OR = 1.008, 95%CI = 1.002-1.014). In contrast with use of preventive care, consumption seemed to play a bigger role than assets when utilizing curative services, particularly for the poorest households (OR_assets _= 0.94, 95%CI = 0.77-1.16; OR_consumption _= 0.61, 95%CI = 0.55-0.68). Both private practice physicians and public practice health personnel had a similar behavior with both curative care outcomes. The density of private practice physicians was also positively associated with *second health problem curative visit *(OR = 1.034, 95%CI: 1.008 - 1.060) after adjusting for predisposing and enabling factors. In contrast, public practice health personnel was negatively related to *second health problem curative visit *(OR = 0.984, 95%CI: 0.973 - 0.996). In this case, however, none of the interaction terms were statistically significant. It is important to highlight that rural households were less likely to use curative care for both the first health problem (OR = 0.76, 95%CI = 0.64-0.89) and the second health problem (OR = 0.64, 95%CI = 0.50-0.82). Similarly, the lack of health insurance was a strong barrier to utilizing both preventive (OR = 0.65, 95%CI = 0.58-0.73) and curative care services (OR = 0.76, 95%CI = 0.71-0.81; OR = 0.71, 95%CI = 0.61-0.81, for first and second reported health problem, respectively).

## Discussion

This study finds evidence of a statistically significant relationship between availability of health providers and utilization of health care services among Ecuadorians. Preliminary ecological analysis showed that provider measures were mostly evenly distributed across provinces. This in turn allowed us to link provider measures at the province level with use of preventive and curative care at the individual level in a multilevel model, to advance our understanding of the context of health care utilization [6,29]. After adjusting for various predisposing, enabling, need factors and interaction terms, density of public practice health personnel was positively associated with use of preventive care, particularly among rural households. In turn, density of private practice physicians was positively associated with use of curative care, particularly among urban households (Table 5).

**Table 5:** Odds Ratios (and 95% confidence intervals) of the association between provider measures and use of preventive and curative care.

		*Curative Care Visits*	
		
Provider measures	Preventive care^a^	First health problem^a^	Second health problem^a^
Public practice physicians	1.045 (0.976 -- 1.119)		
Private practice physicians		1.035 (1.020 -- 1.049)	1.043 (1.017 -- 1.070)
Public practice health personnel	1.009 (0.997 -- 1.021)	0.985 (0.981 -- 0.989)	0.982 (0.973 -- 0.992)
Private practice health personnel	0.990 (0.973 -- 1.008)		

^**a **^Adjusted for predisposing, enabling, need factors, and interaction terms.

There seems to be a drastic difference in the effect of provision of private physician services among rural households. No significant effect was found for curative care of first (OR = 0.987, 95%CI = 0.974-1.001) and second health problem (OR = 0.983, 95%CI = 0.958-1.009). On the other hand, density of public practice health personnel was positively associated with use of preventive and curative care (first health problem), particularly among rural households. This dichotomy confirms our initial concerns regarding the distribution of health services and professionals in Ecuador. As mentioned before, urban areas in Ecuador concentrate 97% of private practice physicians (86% among public practice physicians), and 96% of private practice health personnel work in urban areas (88% among public practice health personnel). However, 39% of the population still lives in rural areas [19]. Although the disparity is wider than global indicators, the lack of health providers in rural and remote areas is a worldwide issue [30,31]. In the case of Ecuador, there is a double burden as reflected by an unequal distribution of providers both between public and private sectors, and between urban and rural areas [32], which may be aggravated by the international migration of health workers [31].

Strengthening the public health care delivery system, then, could significantly impact people's ability to access health care services, particularly for rural households in Ecuador. Dussault and Franceschini (2006) identified five categories of determinants of geographical distribution of health workers: individual, organizational, health care and educational systems, institutional structures, and broader socio-cultural environments. More succinctly, remuneration seems to be a predominant driving force in health workers retention. However, other factors may play an equally important role. For instance, the current model of medical education (mostly urban-based, curative, specialized, hospital-centered) has been found to influence the composition of the workforce and ultimately promote a "cosmopolitan ethics" concerned more with individual success rather than overall public health system improvement [33]. Cuba's Latin American School of Medicine (ELAM), Huish points out, presents an alternative model of medical education in which medical personnel are trained for community-oriented service in marginalized areas. Other similar (albeit more recent) initiatives in Latin America include the Family Health Program in Brazil, the Right to Health Reform in Chile, the *Barrio Adentro *mission and the Integrated Community Physicians Training Program in Venezuela.

What these programs seem to have in common is the combination of redistributive approaches and educational processes associated with the right to health as a guiding principle [34]. In that context, Ecuador recently introduced the so called *Basic Health Care Teams *(EBAS) which intend to work at the community level with a focus on health promotion and disease prevention [2]. Another program (in place since 1970), the one-year obligatory rural medical service for recent graduates, has had mixed results and continues to face many challenges [35].

The following caveats should be considered when interpreting the empirical findings of this study. First, in terms of data availability, this study relied primarily on secondary national survey data (ENDEMAIN 2004). In survey design, an important assumption is that although questions are usually asked about temporal (dynamic) processes, "fixed" populations are studied [36]. Second, the presence of facilities and providers was measured at the province/region level. Such level of aggregation prohibits its use for local decision-making. Besides, it does not account for issues such as overlapping coverage, redundant services, potential for overcrowded facilities, and variation in quality of services [16]. Also, regression model results were susceptible to the modifiable areal unit problem (MAUP) since space was fragmented in administrative province/regions [37,38]. MAUP is present on spatial data and can be defined as the imposition of artificial units (e.g. provinces) on a rather continuous geographical phenomenon, such as density of providers, producing an scale or aggregation problem [21].

Yet, this study analyzed preliminary data at the province level, calculated Moran's I (both globally and locally), which provided empirical evidence of spatial randomness in the geographical distribution of provider measures. Concurring with previous research, spatial visualization of provider measures proved to be an important complement to tabular ecological analysis [39]. Future research should consider utilizing a more "relational" perspective that may reinforce the idea of a reciprocal connection of people-space [40]; and refining the administrative division (probably at the canton or parroquia levels) to facilitate linkages with provider data at those levels [6], and allow more accurate empirical analyses [41-43]. Also, public participation in health care policy planning and evaluation is an issue not addressed in this study. However, it is one that requires further inspection. In Ecuador, the active involvement of health care users in monitoring and evaluation of the health care system is promoted at local health councils. To date, however, no formal evaluation of its effectiveness in advancing health care decision making (including distribution of health facilities and workers) has been conducted. Recent research suggests the need to balance an expert-led process and one "that emphasizes public involvement in decision making" [44]. Public Participation GIS could be an important tool to involve local communities in a more collaborative decision-making process [45].

## Conclusions

This study was one of few attempting to connect use of health services with the context in which utilization occurs, by including health care provider measures. This approach acknowledged the important connections between individual health care utilization behaviors and contextual factors [46]. In other words, this study went beyond the "population at risk" perspective of the original Andersen's model, to assess delivery system measures that allowed us to contrast utilization with provision of services [6,8]. For Latin America, this study adds to recent empirical work on the context of health care utilization [16], by combining preliminary ecological analysis (at the province level) with a multilevel regression framework. Finally, the present study supports the rationale for building stronger national health care and health information systems [16,42]. Such an infrastructure could improve people's access to health care and more transparently inform the public and policymakers on the advances of health care reform in Ecuador and other low- and middle-income countries [32,47].

## Competing interests

The authors declare that they have no competing interests.

## Authors' contributions

DFLC conceptualized the study, measures, and conducted all data analyses presented. CC supported the study conceptualization, measures, and provided editorial comments and suggestions on the interpretation of findings. Both authors read and approved the final manuscript.

## Pre-publication history

The pre-publication history for this paper can be accessed here:

http://www.biomedcentral.com/1472-6963/10/64/prepub
